# Supplementary material for: Architecture and activation of human muscle phosphorylase kinase
Source: Nat Commun. 2024 Mar 28;15:2719. doi: 10.1038/s41467-024-47049-2 (PMC10978961; doi:10.1038/s41467-024-47049-2)
Supplement: Supplementary file 1 — Supplementary Information [file 41467_2024_47049_MOESM1_ESM.pdf]

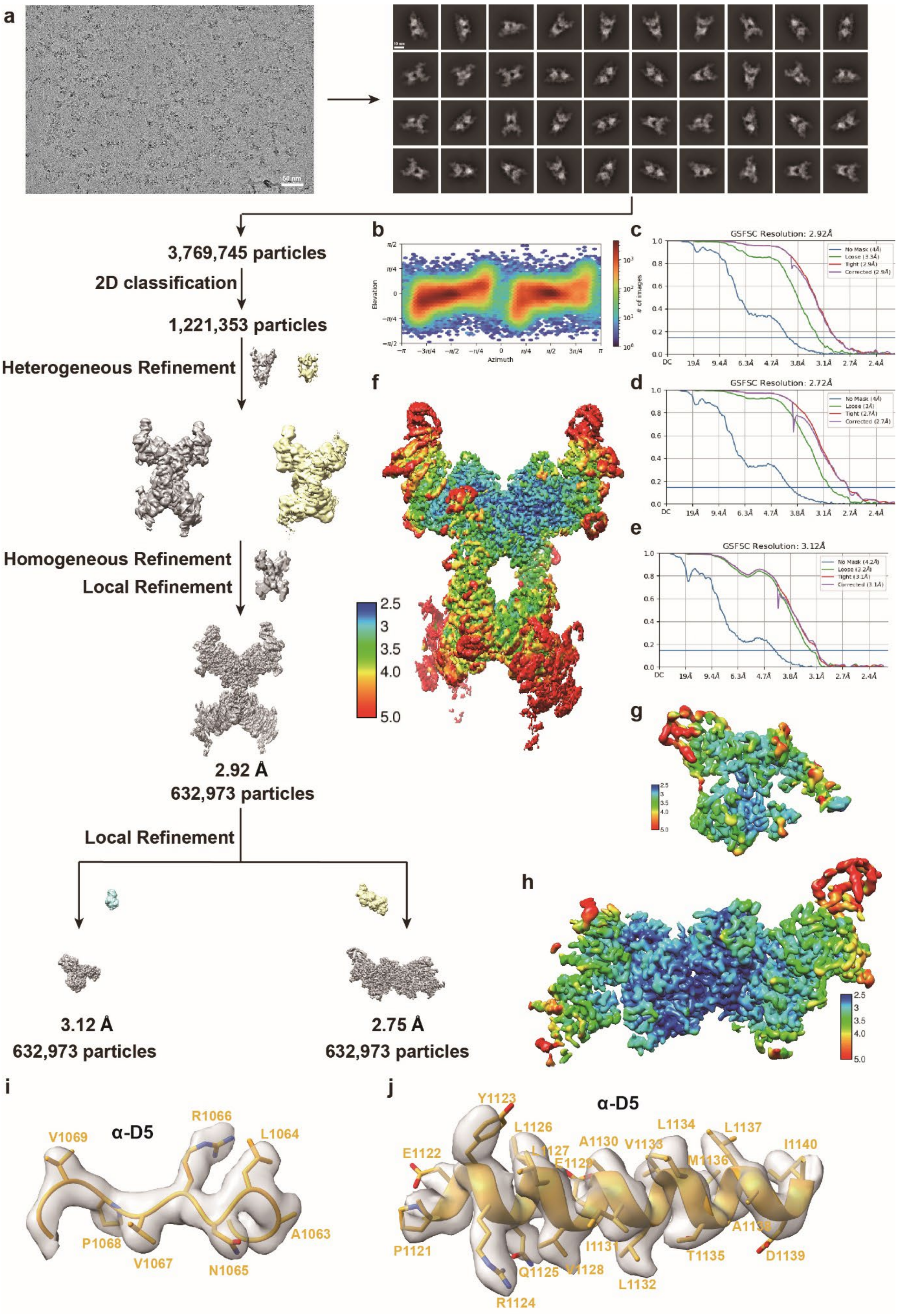

a
50 nm
3,769,745 particles
2D classification
1,221,353 particles
Heterogeneous Refinement
Homogeneous Refinement
Local Refinement
2.92 Å
632,973 particles
Local Refinement
3.12 Å
632,973 particles
2.75 Å
632,973 particles
b
Elevation
Azimuth
# of images
c
GSFSC Resolution: 2.92Å
No Mask (4Å)
Loose (3.3Å)
Tight (2.9Å)
Corrected (2.9Å)
d
GSFSC Resolution: 2.72Å
No Mask (4Å)
Loose (3Å)
Tight (2.7Å)
Corrected (2.7Å)
e
GSFSC Resolution: 3.12Å
No Mask (4.2Å)
Loose (3.2Å)
Tight (3.1Å)
Corrected (3.1Å)
DC 19Å 9.4Å 6.3Å 4.7Å 3.8Å 3.1Å 2.7Å 2.4Å
f
2.5
3
3.5
4.0
5.0
g
h
i
α-D5
V1069
R1066
L1064
P1068
V1067
N1065
A1063
j
α-D5
Y1123
L1126
L1127
A1130
V1133
L1134
L1137
I1140
E1122
E1129
M1136
P1121
I1131
A1138
T1135
D1139
Q1125
V1128
L1132
R1124

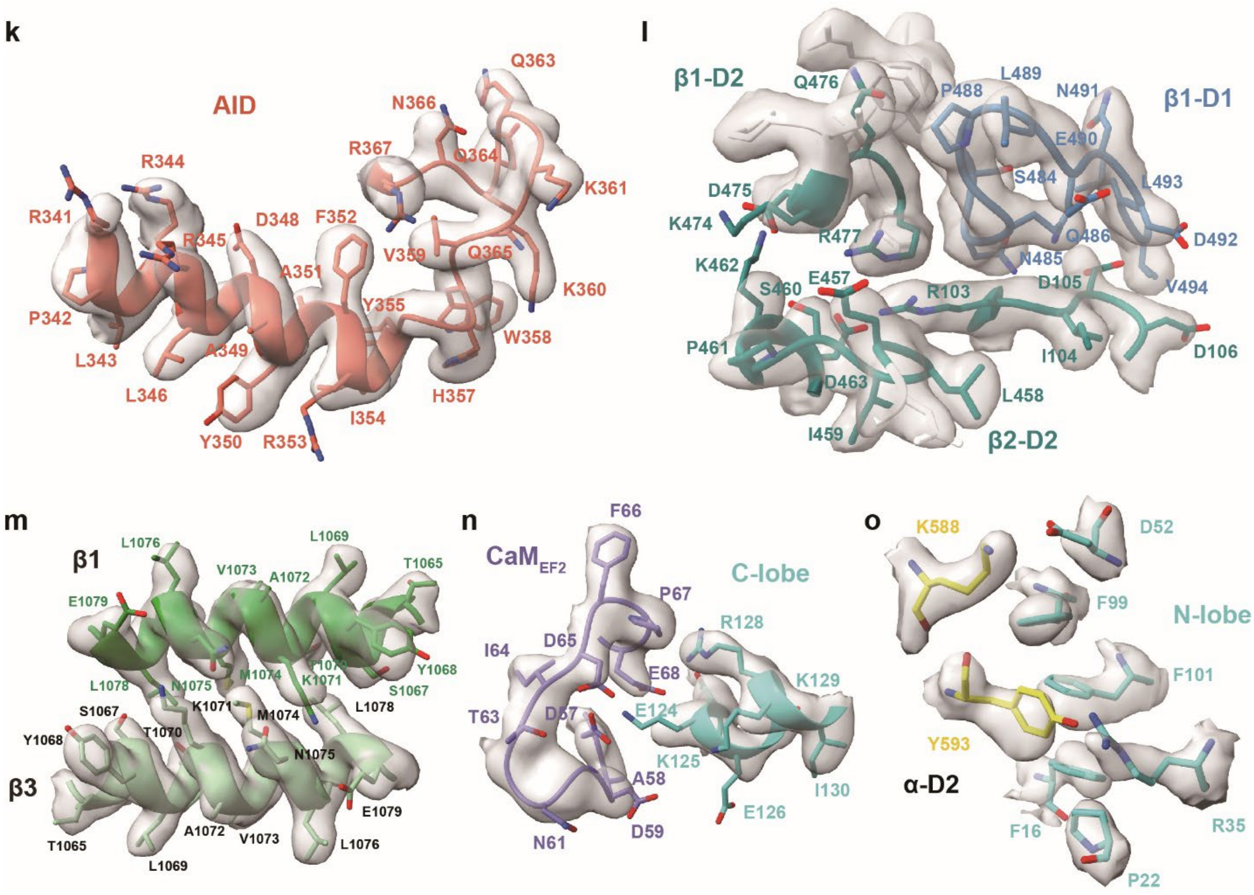

Supplementary Figure 1. Cryo-EM 3D reconstruction of the inactive PhK complex.

a. Flowchart of cryo-EM data processing.

b. Angular particle distribution heat map.

c–e. Gold-standard Fourier shell correlation (GSFSC) curves of the inactive PhK holoenzyme, αβγδ subcomplex, and γδ subcomplex, respectively.

f–h. Resolution estimations for the final maps of the inactive human muscle PhK holoenzyme, αβγδ subcomplex, and γδ subcomplex, respectively.

i–o. EM densities for representative regions in α-D5, AID, β1/β2 interface, β1/β3 interface, EF2/C-lobe interface, and KD/α-D2 interface, respectively.

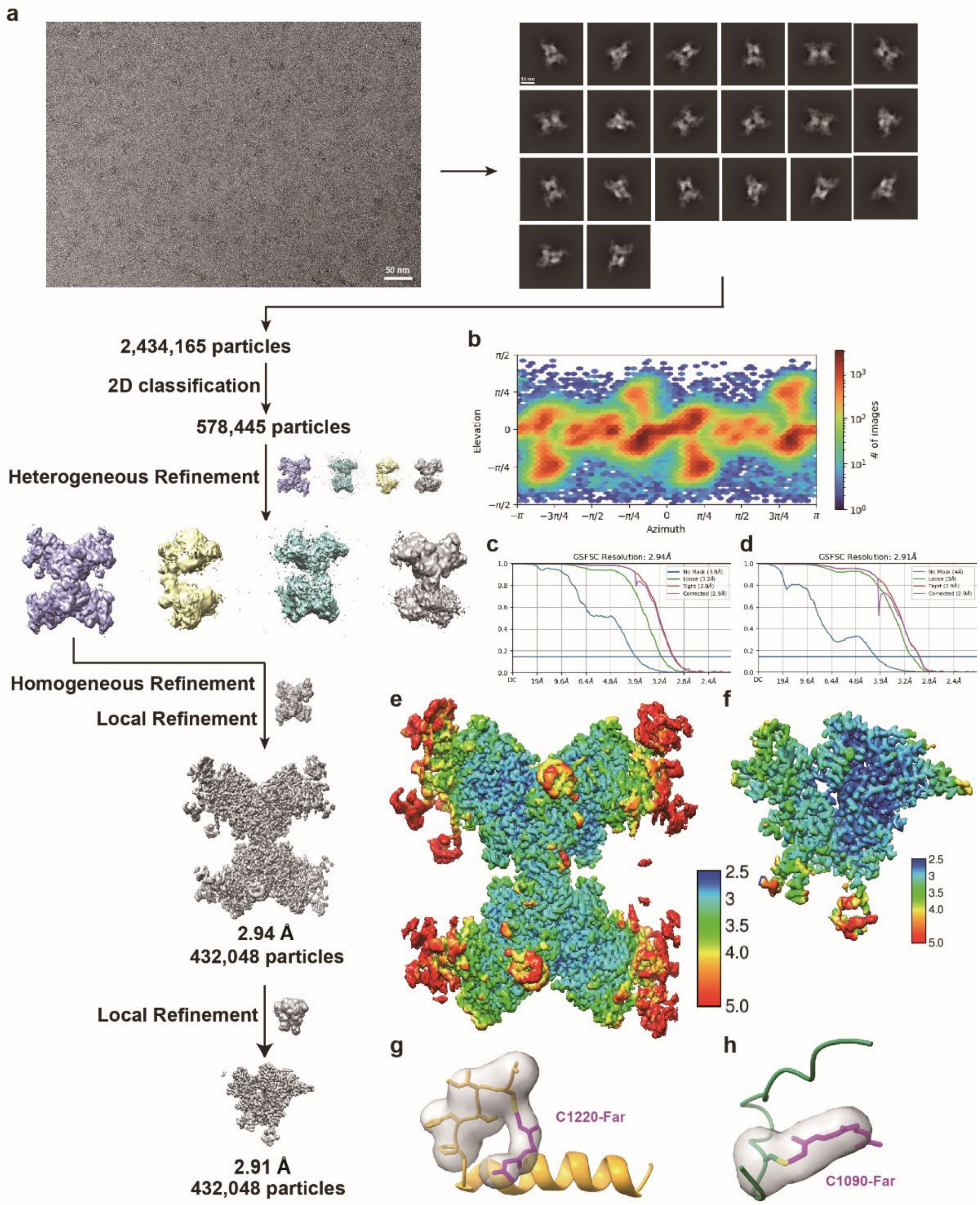

Supplementary Figure 2. Cryo-EM 3D reconstruction of the active PhK complex.

a. Flowchart of cryo-EM data processing.

b. Angular particle distribution heat map.

c, d. Gold-standard Fourier shell correlation (GSFSC) curves of the active PhK and the αγ subcomplex, respectively.

e, f. Resolution estimations for the final maps of the active PhK and the αγ subcomplex, respectively.

g. Density map for the farnesyl group on the α-subunit.

h. Density map for the farnesyl group on the β-subunit.

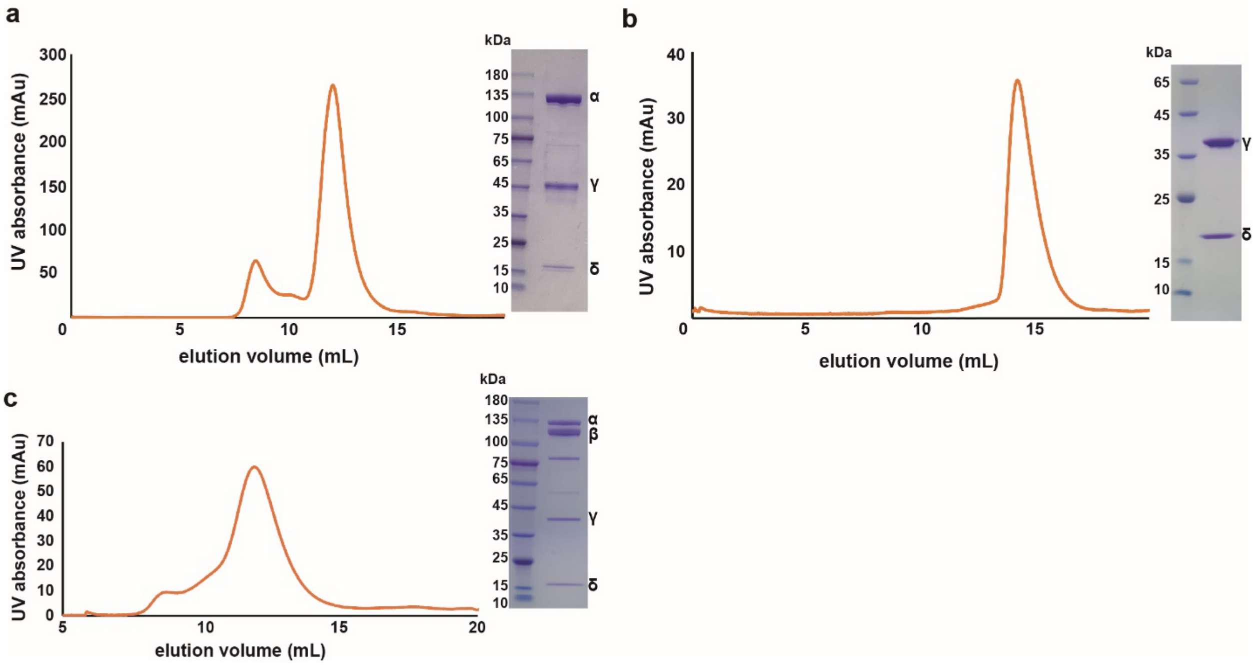

Supplementary Figure 3. Purification of the αγδ, $\gamma_{326}\delta$ subcomplexes, and active PhK holoenzyme.

a. Size-exclusion chromatography and SDS-PAGE analysis of the αγδ complex.

b. Size-exclusion chromatography and SDS-PAGE analysis of the $\gamma_{326}\delta$ complex.

c. Size-exclusion chromatography and SDS-PAGE analysis of the active PhK holoenzyme.

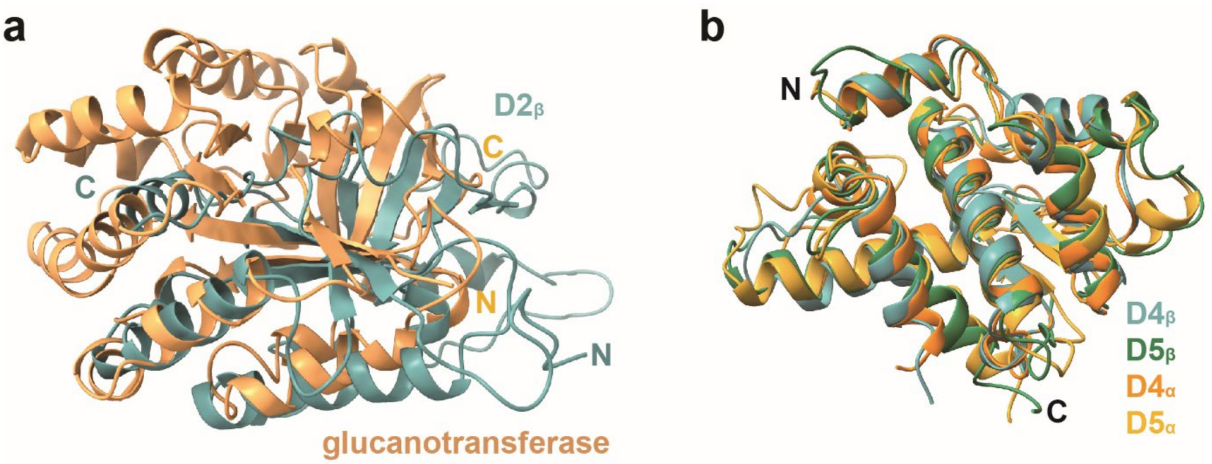

Supplementary Figure 4. The D2, D4, and D5 domains of the α- and β-subunits.

a. Structural overlay of the D2 domain of the β-subunit and a glucanotransferase (PDB: 1K1Y), shown in cyan and yellow, respectively.

b. Structural overlay of the D4 and D5 domains of the α- and β-subunits.

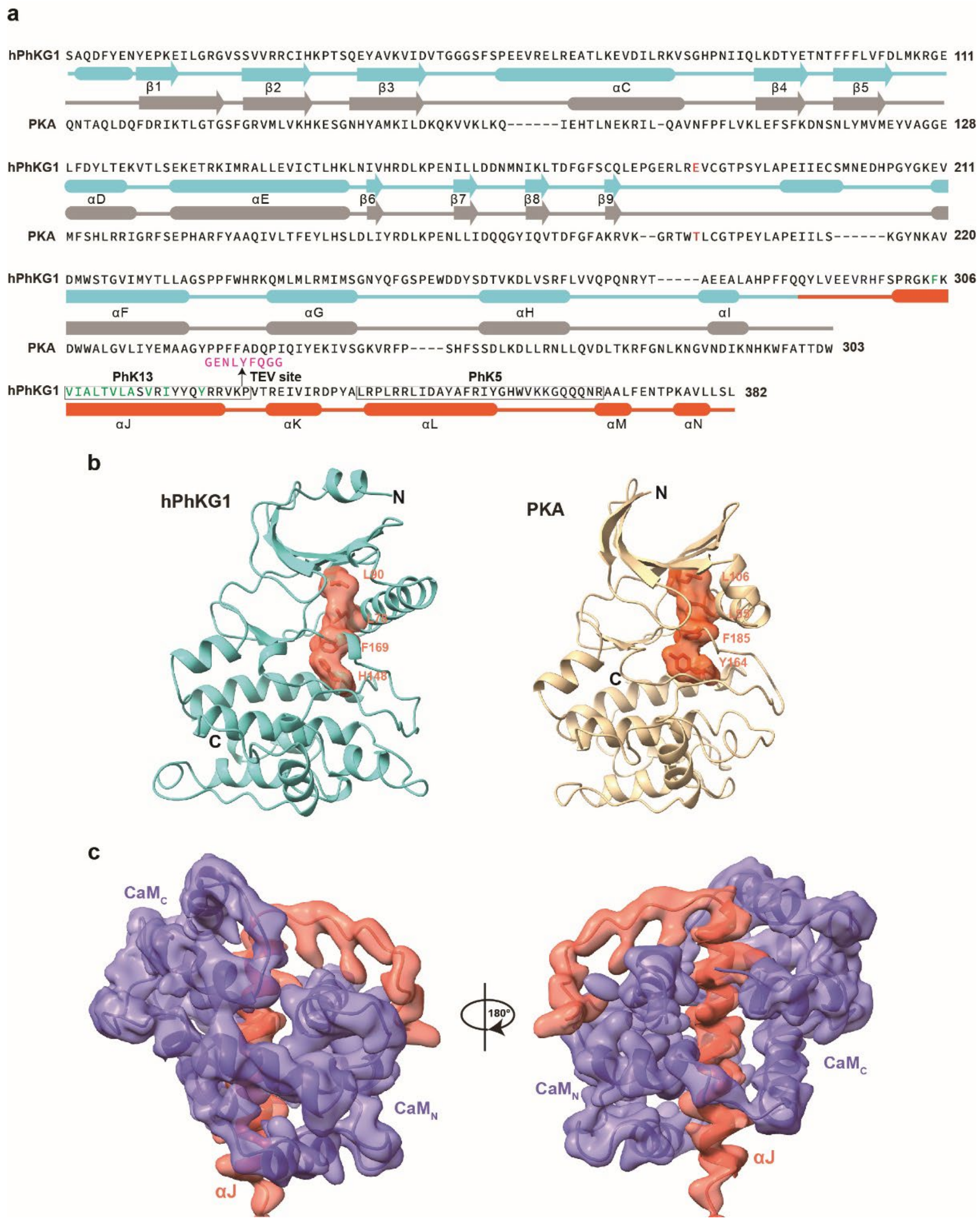

Supplementary Figure 5. Structure of the γ-subunit and its interaction with calmodulin.

a. Sequence alignment between the γ-subunit and PKA. The secondary structures of the two proteins are indicated. Glu183$_{\gamma}$ and Thr197$_{\mathrm{PKA}}$ are highlighted in red. The TEV protease cleavage site (GENLYFQGG) is introduced between Lys325 and Pro326 of the γ-subunit in the $\alpha\gamma_{\mathrm{TEV}}\delta$ mutant. The PhK13 and PhK5 peptide regions are highlighted using black boxes.

b. Leu78$_{\gamma}$, Leu90$_{\gamma}$, His148$_{\gamma}$, and Phe169$_{\gamma}$form an intact "regulatory spine" in the γ-subunit. The "regulatory spine" of PKA is shown on the right for comparison.

c. The cryo-EM density map of αJ–calmodulin region shown in two orientations.
