# Supplementary material for: Architecture and activation of human muscle phosphorylase kinase
Source: Nat Commun. 2024 Mar 28;15:2719. doi: 10.1038/s41467-024-47049-2 (PMC10978961; doi:10.1038/s41467-024-47049-2)
Supplement: Supplementary file 3 — Description of Additional Supplementary Files [file 41467_2024_47049_MOESM3_ESM.pdf]

Description of Additional Supplementary Materials

File Name: Supplementary Movie 1

Description: Computational morphing illustrates how ADP could allosterically regulate PhK activity. The interaction between D2β and D2α forms the α1/β2 and α2/β1 dimers, while D2α also interacts with the KD Nlobe to maintain the inactive state of the γ-subunit. ADP triggers a conformational change in D2β, potentially leading to the displacement of D2α, thereby contributing to disrupting the α-subunit–KD interaction. The inactive structure is depicted in grey, and the D2β and D2α domains from the inactive state are morphed into the active structure, highlighted in blue and yellow.

File Name: Supplementary Data 1

Description: List of primers used in the study and the mass spectrometry data.
