# Supplementary material for: Architecture and activation of human muscle phosphorylase kinase
Source: Nat Commun. 2024 Mar 28;15:2719. doi: 10.1038/s41467-024-47049-2 (PMC10978961; doi:10.1038/s41467-024-47049-2)
Supplement: Supplementary file 6 — Reporting Summary [file 41467_2024_47049_MOESM6_ESM.pdf]

Corresponding author(s): Junyu Xiao
Last updated by author(s): Feb 20, 2024

Reporting Summary

Nature Portfolio wishes to improve the reproducibility of the work that we publish. This form provides structure for consistency and transparency in reporting. For further information on Nature Portfolio policies, see our Editorial Policies and the Editorial Policy Checklist.

Statistics

For all statistical analyses, confirm that the following items are present in the figure legend, table legend, main text, or Methods section.

n/a	Confirmed	
☐	☒	The exact sample size (*n*) for each experimental group/condition, given as a discrete number and unit of measurement
☐	☒	A statement on whether measurements were taken from distinct samples or whether the same sample was measured repeatedly
☒	☐	The statistical test(s) used AND whether they are one- or two-sided<br>*Only common tests should be described solely by name; describe more complex techniques in the Methods section.*
☒	☐	A description of all covariates tested
☒	☐	A description of any assumptions or corrections, such as tests of normality and adjustment for multiple comparisons
☐	☒	A full description of the statistical parameters including central tendency (e.g. means) or other basic estimates (e.g. regression coefficient) AND variation (e.g. standard deviation) or associated estimates of uncertainty (e.g. confidence intervals)
☒	☐	For null hypothesis testing, the test statistic (e.g. *F*, *t*, *r*) with confidence intervals, effect sizes, degrees of freedom and *P* value noted<br>*Give P values as exact values whenever suitable.*
☒	☐	For Bayesian analysis, information on the choice of priors and Markov chain Monte Carlo settings
☒	☐	For hierarchical and complex designs, identification of the appropriate level for tests and full reporting of outcomes
☒	☐	Estimates of effect sizes (e.g. Cohen's *d*, Pearson's *r*), indicating how they were calculated

Our web collection on statistics for biologists contains articles on many of the points above.

Software and code

Policy information about availability of computer code

Data collection	Cryo-EM data collection: EPU version 2.1.2 (Thermo Fisher Scientific).<br>For glucoamylase assay: The absorbance was measured at 540 nm using the BioTek Cytation 5 (Agilent) in 96-well plate.<br>For Mass spectrometry: The sample was analyzed by LC-MS/MS using the Orbitrap Fusion™ Lumos™ Tribrid™ mass spectrometer (Thermo Fisher Scientific).
Data analysis	cryoSPARC version 3.2.1 and deepEMhancer were used to process the cryo-EM data.<br>The local resolution map was analyzed using cryoSPARC version 3.2.1 and displayed using UCSF ChimeraX version 1.5rc202211120143.<br>Structural modeling and refinement were performed using Coot version 0.9.4.1 and Phenix version 1.19.<br>MS/MS spectra were searched using Proteome Discoverer software (version 2.1) with a UniportKB human database.<br>The glucoamylase assay data were analyzed with Prism 9.0.0 (GraphPad).<br>Initial model of α, β and γ-subunit were generated by Alphafold2.

For manuscripts utilizing custom algorithms or software that are central to the research but not yet described in published literature, software must be made available to editors and reviewers. We strongly encourage code deposition in a community repository (e.g. GitHub). See the Nature Portfolio guidelines for submitting code & software for further information.

Data

Policy information about availability of data

All manuscripts must include a data availability statement. This statement should provide the following information, where applicable:

- Accession codes, unique identifiers, or web links for publicly available datasets
- A description of any restrictions on data availability
- For clinical datasets or third party data, please ensure that the statement adheres to our policy

Cryo-EM density maps of PhK have been deposited in the Electron Microscopy Data Bank with accession codes EMD-36212 (inactive, overall holoenzyme), EMD-36214 (inactive, αβγδ subcomplex), EMD-36215 (inactive, γδ subcomplex), EMD-36213 (Ca2+, overall holoenzyme), and EMD-36216 (Ca2+, αγ subcomplex). Structural coordinates have been deposited in the Protein Data Bank with the accession codes 8JFK (inactive, holoenzyme), 8XYA (inactive, αβγδ subcomplex), 8XYB (inactive, γδ subcomplex), 8JFL (Ca2+, holoenzyme) and 8XY7 (Ca2+, αγ subcomplex). We have also referenced in this work PDB accessions 1CDL, 5Z3F, 2PHK, and 1K1Y. Uncropped gels and blots underlying Figures 1b-c, 2, 3f and Supplementary Figure 3b are provided in the Source Data file.

Research involving human participants, their data, or biological material

Policy information about studies with human participants or human data. See also policy information about sex, gender (identity/presentation), and sexual orientation and race, ethnicity and racism.

Reporting on sex and gender	N/A
Reporting on race, ethnicity, or other socially relevant groupings	N/A
Population characteristics	N/A
Recruitment	N/A
Ethics oversight	N/A

Note that full information on the approval of the study protocol must also be provided in the manuscript.

Field-specific reporting

Please select the one below that is the best fit for your research. If you are not sure, read the appropriate sections before making your selection.

☒ Life sciences ☐ Behavioural & social sciences ☐ Ecological, evolutionary & environmental sciences

For a reference copy of the document with all sections, see nature.com/documents/nr-reporting-summary-flat.pdf

Life sciences study design

All studies must disclose on these points even when the disclosure is negative.

Sample size	No any statistical methods were used to predetermine the sample size.<br>For cryo-EM structure determination: 3,827 movies in inactive state and 4,770 movies in active state were collected. The number of particles used for EM reconstructions has been stated in the processing workflow.
Data exclusions	Junk micrographs and particles were removed following the standard procedures in cryoSPARC, because it is essentially to optimize the best particles for high-resolution map generation.
Replication	Each kinase assay has been repeated three times. The TEV cleavage pull-down assay and glucoamylase assay have also been repeated three times. All attempts at replication were successful.
Randomization	For cryo-EM data processing, the datasets were randomly split into two halves for structure determination.<br>Randomization is unnecessary and not applicable for the biochemical assays in this study.
Blinding	Blinding is unnecessary and not applicable for the biochemical assays and cryo-EM structure determination in this study.

Reporting for specific materials, systems and methods

We require information from authors about some types of materials, experimental systems and methods used in many studies. Here, indicate whether each material, system or method listed is relevant to your study. If you are not sure if a list item applies to your research, read the appropriate section before selecting a response.

Materials & experimental systems

n/a	Involved in the study	
☐	☒	Antibodies
☐	☒	Eukaryotic cell lines
☒	☐	Palaeontology and archaeology
☒	☐	Animals and other organisms
☒	☐	Clinical data
☒	☐	Dual use research of concern
☒	☐	Plants

Methods

n/a	Involved in the study	
☒	☐	ChIP-seq
☒	☐	Flow cytometry
☒	☐	MRI-based neuroimaging

Antibodies

Antibodies used	anti-PYGL (phospho S15) antibody (Abcam, ab227043) and goat Anti-rabbit IgG HRP (HS101-01).
Validation	Rabbit Recombinant Monoclonal PYGL phospho S15 antibody was validated in IP, Dot, WB, IHC-Fr, IHC-P and tested in Mouse, Rat, Human samples according to the manufacturer's website and it has also been validated by the kinase assay shown in Figure 1c and Figure 5b.

Eukaryotic cell lines

Policy information about cell lines and Sex and Gender in Research

Cell line source(s)	HEK293F cells were originally purchased from ATCC.
Authentication	The cell lines were not authenticated.
Mycoplasma contamination	The cells were regularly tested for free of Mycoplasma contamination by standard PCR method.
Commonly misidentified lines (See ICLAC register)	No commonly misidentified lines were used.

Plants

Seed stocks	N/A
Novel plant genotypes	N/A
Authentication	N/A
